# Diverse Effects of β-Carotene on Secretion and Expression of VEGF in Human Hepatocarcinoma and Prostate Tumor Cells

**DOI:** 10.3390/molecules17043981

**Published:** 2012-04-02

**Authors:** Huei-Yan Chen, Shu-Ming Huang, Chih-Min Yang, Miao-Lin Hu

**Affiliations:** Department of Food Science and Biotechnology, National Chung Hsing University, 250 Kuo Kuang Road, Taichung 402, Taiwan

**Keywords:** B16F10, β-carotene, PC-3, SK-Hep-1, VEGF

## Abstract

Oral administration of β-carotene (BC) was found to exert opposite effects on plasma levels of vascular endothelial growth factor (VEGF) in two animal models. One study in nude mice injected via tail vein with hepatocarcinoma SK-Hep-1 cells showed that BC decreases the plasma VEGF level, whereas the other study in nude mice injected subcutaneously with prostate tumor PC-3 cells showed that BC increases the plasma VEGF level. Herein we investigated whether BC (0.5–20 μM) possesses diverse effects on VEGF secretion in SK-Hep-1, PC-3 and melanoma B16F10 cells. We found that incubation of SK-Hep-1 cells with BC (1–20 μM) for 6 h significantly decreased VEGF secretion, whereas BC (1–10 μM) significantly increased the VEGF secretion in PC-3 cells. However, these effects disappeared at 12 h of incubation. Similar effects occurred in VEGF mRNA and protein expression after treatment of SK-Hep-1 and PC-3 cells with BC for 6 h. In contrast, BC (0.5–20 μM) did not affect mRNA and protein expression and secretion of VEGF in B16F10 cells. We also found that the proliferation of SK-Hep-1 and B16F10 cells was significantly inhibited by 20 μM BC at 6 and 12 h of incubation, whereas the proliferation of PC-3 cells was significantly inhibited by 20 μM BC at 12 h of incubation. In summary, the present study demonstrated the tumor-specific effect of BC on VEGF secretion in different cancer cell lines.

## 1. Introduction

The role of β-carotene (BC) on angiogenesis is contradictory. Two studies have indicated that BC possesses pro-angiogenic activity in endothelial cells and in matrigel-plug model [1,2]. One is that BC (3.0 μM) accelerates the basic fibroblast growth factor-induced tube formation and migration of endothelial cells as well as development of microcapillaries in mice with matrigel plug injection subcutaneously [1]. The other is that BC supplementation (12,000 mg/kg) in the diet to Balb/c mice with Matrigel-plug transplantation exhibits pro-angiogenic activity [2]. In contrast, one study has indicated that BC exhibits anti-angiogenic activity by inhibition of B16F10 melanoma-induced neovascularization *in vivo* and inhibition of proliferation, migration and tube formation in endothelial cells *in vitro* [3]. 

Vascular endothelial growth factor (VEGF), an endothelium-specific secreted protein, plays an important role in angiogenesis [4]. Previously, we observed that oral supplementation of BC decreases plasma VEGF levels in nude mice transplanted with hepatocarcinoma SK-Hep-1 cells [5] but increases plasma VEGF in nude mice transplanted with prostate tumor PC-3 cells [6]. Still another study indicated that BC administration (i.p.) inhibit tumor-specific neovascularization that involved reduction of VEGF expression in lung tissues of B16F10-bearing C57BL/6 mice [3]. Based on these conflicting findings, we hypothesized that the effects of BC on VEGF expression may be tumor-specific. To test this hypothesis, we investigated the effects of BC on VEGF secretion and mRNA and protein expression in different cancer cell lines including human hepatocarcinoma SK-Hep-1 cells, prostate tumor PC-3 cells, and melanoma B16F10 cells.

## 2. Results and Discussion

**Figure 1:**
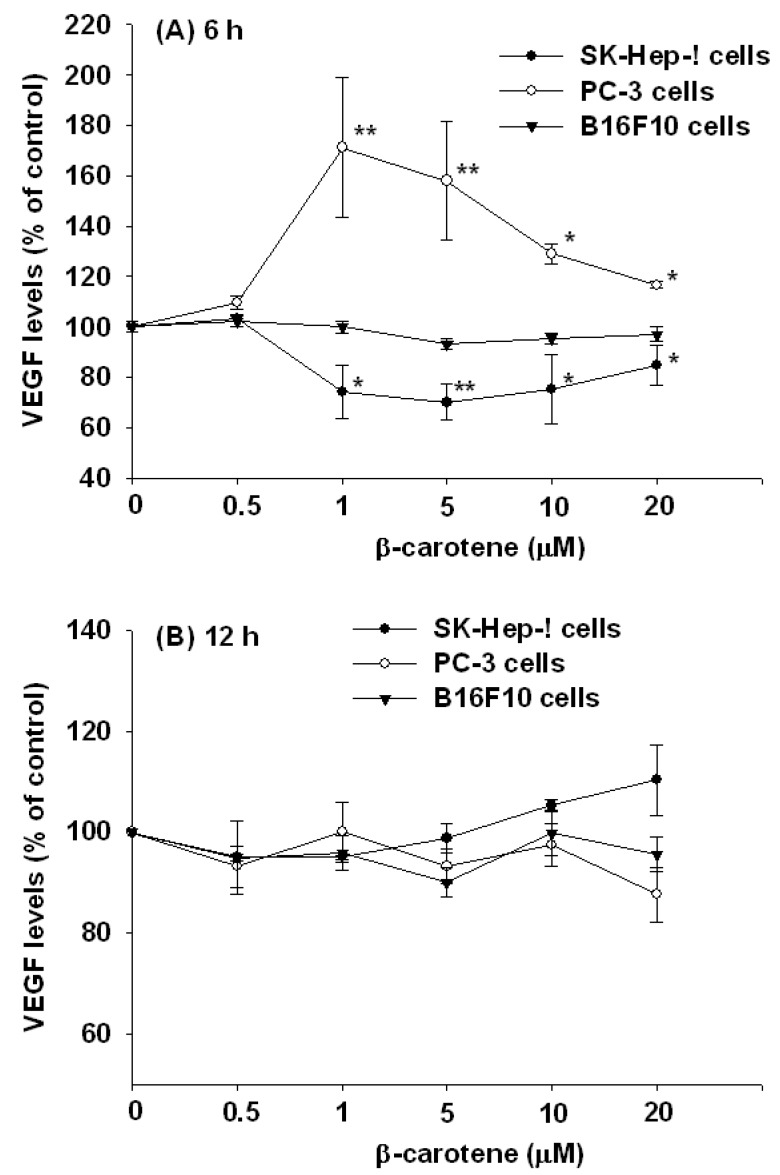
Effect of β-carotene (BC) on VEGF secretion in SK-Hep-1, PC-3 and B16F10 cells. Cells were incubated with BC (0–20 μM) for 6 and 12 h. (**A**) 6 h; (**B**) 12 h. Data (means ± SD) are from three or four separate experiments; * *P* < 0.05; ** *P* < 0.01 compared to control (0 μM β-carotene).

Literature reports on the effects of BC on tumor angiogenesis and expression of VEGF are contradictory. Surprisingly, two recent studies of ours demonstrated opposite effects of oral supplementation of BC on plasma VEGF levels in nude mice transplanted with either hepatocarcinoma or prostate tumor cells [5,6]. To resolve this issue, we investigated the effects of BC on VEGF secretion and mRNA and protein expression* in vitro* in three different cancer cell lines, including hepatocarcinoma SK-Hep-1 cells, prostate tumor PC-3 cells and melanoma B16F10 cells. The main finding was that BC (1–20 μM) significantly decreased VEGF secretion and mRNA and protein expression in SK-Hep-1 cells at 6 h of incubation, and this effect was U-shaped,* i.e.*, the strongest inhibition (30% for secretion, *P* < 0.01; 49% for mRNA, *P* < 0.01 and 32% for protein, *P *< 0.01) occurred at 5 μM BC (Figure 1–Figure 3). In contrast, BC (1–10 μM) markedly increased VEGF secretion in PC-3 cells in a bell-shaped manner at 6 h of incubation,* i.e.*, the highest increase (71% for secretion, *P* < 0.01; 327% for mRNA, *P* < 0.01 and 191% for protein, *P* < 0.01) was at 1 μM BC (Figure 1–Figure 3). The opposite effects of BC on VEGF secretion in SK-Hep-1 and PC-3 cells appeared to be transient, as they disappeared at 12 h of incubation (Figure 1). Nevertheless, these results appear to support our previous findings that oral supplementation of BC decreases the plasma VEGF levels in nude mice xenografted with SK-Hep-1 cells via tail vein [5] but increases the plasma VEGF levels in nude mice injected with PC-3 cells subcutaneously [6]. It is noteworthy that in these two experiments, the effective concentrations (5 and 1 μM) of BC are physiological or supra-physiological, as compared to the plasma BC concentration of 0.56 μM in PC-3-bearing nude mice orally supplemented with relatively high BC (16 mg/kg twice a week) for 7 week [6] or the plasma BC levels which increased 14-fold from 0.48 μM to 6.83 μM in healthy older woman after supplementation with 90 mg/d β-carotene for 3 weeks [7].

An* in vivo* study has shown that BC administration (60 mg/kg body weight; i.p., 5 times during a 24-h period) inhibits the neovascularization induced by the melanoma B16F10 cells, suggesting that this effect is, at least in part, associated with the inhibition of VEGF mRNA expression in lung tissues [3]. However, we found in the present study that BC (0.5–20 μM) did not affect the secretion and mRNA and protein expression of VEGF in melanoma B16F10 cells at 6 and 12 h of incubation (Figure 1–Figure 3). It is unclear whether B16F10 cells treated with BC for longer than 12 h may result in inhibition of VEGF secretion, as BC has been shown to be unstable* in vitro*, and it degrades rapidly in cell culture media (~35% degradation at 24 h of incubation) [8]. 

**Figure 2:**
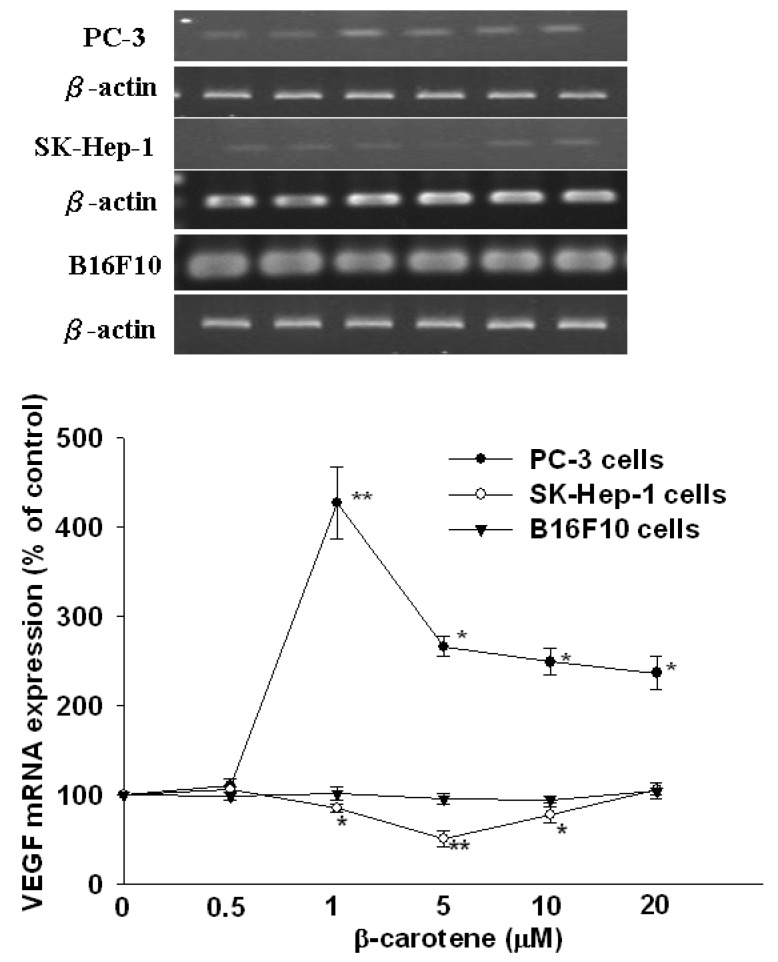
Effect of β-carotene (BC) on mRNA expression of VEGF in SK-Hep-1, PC-2 and B16F10 cells. Cells were incubated with BC (0–20 μM) for 6 h. Data (means ± SD) are from three or four separate experiments; * *P* < 0.05; ** *P* < 0.01 compared to control (0 μM β-carotene).

**Figure 3:**
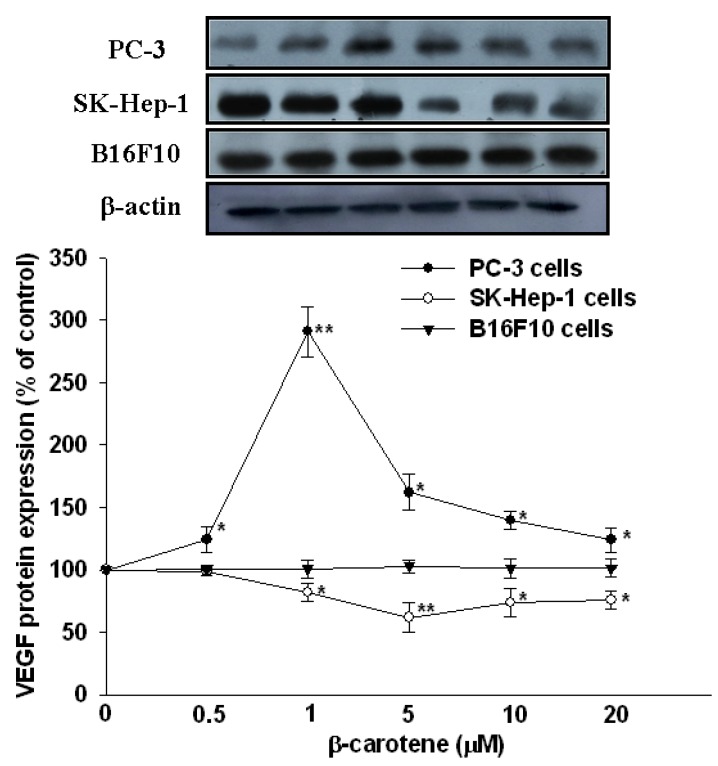
Effect of β-carotene (BC) on protein expression of VEGF in SK-Hep-1, PC-3 and B16F10 cells. Cells were incubated with BC (0–20 μM) for 6 h. Data (means ± SD) are from three or four separate experiments; * *P* < 0.05; ** *P* < 0.01 compared to control (0 μM β-carotene).

**Figure 4:**
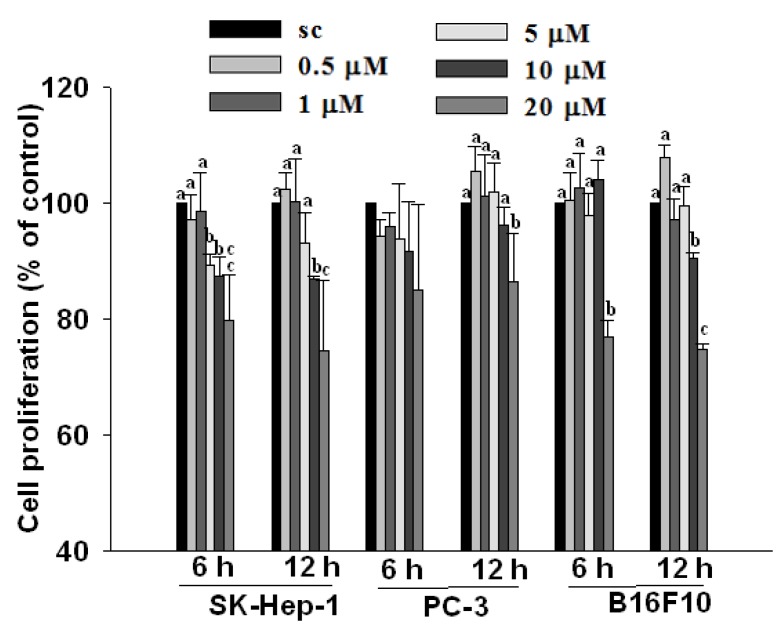
Effect of β-carotene (BC) on cell proliferation of SK-Hep-1, PC-3 and B16F10 cells. Cells were incubated with BC (0–20 µM) for 6 and 12 h. Data (means ± SD) are from three or four separate experiments; means for each cell type measured at the same incubation time without a common alphabetic letter differ significantly (*P* < 0.05).

Although BC added at 20 μM decreased cell proliferation of all three types of cancer cells, there was no significant inhibition on cell proliferation when BC added at concentrations lower than or equal to 5 µM (Figure 4). In addition, BC added at concentrations ≤5 μM with an incubation time of 6 h either increased (in PC-3 cells) or decreased (SK-Hep-1 cells) VEGF secretion and mRNA and protein expression (Figure 1–Figure 3). These results suggest that the modulating effect of BC on VEGF levels in SK-Hep-1 and PC-3 cells is not related to inhibition of cell proliferation.

The reason for the U-shaped concentration effect (for SK-Hep-1) and the bell-shaped concentration effect (for PC-3 cells) on VEGF secretion and mRNA expression is unclear. A possible explanation is that, at higher cellular concentrations, the antioxidant property of some carotenoids may shift to prooxidant property [9]. Indeed, some studies have shown that BC exhibits lowered effectiveness as antioxidants and anticarcinogens in cell culture studies at concentrations >10 μM [9,10].

In conclusion, the diverse effects of BC on VEGF secretion in cancer cell lines appear to be tumor-specific. *In vivo* studies that are carefully designed to elucidate the diverse effects are needed to confirm the present findings.

## 3. Experimental

### 3.1. Reagents and β-Carotene Preparation

All chemicals used are of the highest grade. Tetrahydrofuran (THF) and butylated hydroxytoluene (BHT) were purchased from Sigma (St. Louis, MO, USA). Dulbecco’s Modified Eagle Medium (DMEM), RPMI1640, nonessential amino acid, penicillin/streptomycin, sodium pyruvate, fetal bovine serum (FBS) and trypsin were obtained from Gibco/BRL (Grand Island, NY, USA). β-Carotene was purchased from Wako (Osaka, Japan), and soluble in THF/BHT to form stock solution of 10 mM, which was diluted with THF at 1:1, 1:3, 1:19 and 1:39 ratio, and then diluted with FBS at 1:9 ratio [11]. THF/BHT-FBS-β-carotene was added to the culture medium at a calculated final concentration of 0.5, 1, 5, 10 or 20 µM. THF at 0.2% (v/v) and FBS at 1.8% (v/v) served as the solvent control for beta-carotene, which did not significantly affect the assays described below.

### 3.2. Cell Culture and Cell Proliferation Assay

The human hepatoma SK-Hep-1 cell line (BCRC No. 67005), the mouse skin melanoma B16F10 cell line (BCRC No. 60031) and the human prostate carcinoma PC-3 cell line (BCRC No. 67005) were purchased from the Food Industry Research and Development Institute (HsinChu, Taiwan). SK-Hep-1 cells and B16F10 cells were grown in DMEM medium and PC-3 cells were incubated in RPMI medium containing 10% (v/v) fetal bovine serum (FBS), 0.37% (w/v) NaHCO_3_, penicillin (100 U/mL) and streptomycin (100 U/mL) in incubator under 5% CO_2_ and 95% air at 37 °C. For the cell proliferation assay, cells were cultured in 24-well plates at 5 × 10^4^ cell/mL containing 0–20 μM β-carotene in 1 mL serum-free medium for 6, 12 h at 37 °C. Then, the cells were washed with PBS and added 1 mL of 0.5 mg/mL 3-(4,5-dimethylthiazolyl-2)-2,5-diphenyltetrazolium bromide (MTT) to incubate for 1 h at 37 °C. The medium was discarded, and the formazan was dissolved in DMSO and measured spectrophotometrically at 570 nm. The percentage of viable cells was estimated by comparing with untreated control cells. 

### 3.3. Determination of VEGF Secretion in Cultured Cancer Cells

SK-Hep-1, PC-3 and B16F10 cells were treated with β-carotene (0–20 μM) for 6 and 12 h in serum free medium. VEGF levels in the medium were determined using ELISA commercial kit (Endogen Human ELISA Kit, Pierce Biotechnology, Inc., in Rockford, IL, USA) at a wavelength of 450 nm. The assay was performed in three times by following the instruction of the manufacturer.

### 3.4. RNA Extraction and Reverse-transcriptase PCR

Cellular total RNA was extracted with REzol reagent (Protech, Taipei, Taiwan), and 1 µg of total RNA was reverse-transcribed by using oligo-dT as a primer in 20 µL reverse-transcription solutions (PROMEGA, USA). The RT-PCR conditions for VEGF and β-actin were as follows: initial denaturation at 95 °C for 1 min, and 1 min annealing time (55 °C for VEGF and 60 °C for β-actin), 1 min amplification time for 40 cycles after an activation step of 2 min at 95 °C. The PCR products were added 6× staining buffer (EZVISION THREE DNA DYE & BUFFER, Amresco, USA) and were subjected to 1% agarose gel electrophoresis. The primers used in this study were as follows: VEGF forward 5′-AGGAGGAGGGCAGAATCATCA-3′, reverse 5′-TCTCGATTGGATGGCAGTAGC-3′; β-actin forward 5′-GTGGGGCGCCCCAGGCACCA-3′, reverse 5′-CACCCCGCGGGGTCCGTGGT-3′. Matrox Inspector 2.1 software was used to quantify the relative level of VEGF.

### 3.5. Western Blotting

Protein expression of VEGF was measured by western blotting. Cells were incubated with BC (0–20 µM) for 6 h and the medium was removed and then cells were rinsed with PBS twice. After the addition of 0.5 mL of cold RIPA buffer and protease inhibitors cocktail, cells were scraped followed by a vortex at 4 °C for 20 min. The cell lysates were then subjected to a centrifugation of 12,000 × g for 30 min at 4 °C. An amount of protein (40 µg) from the supernatant was resolved by SDS-PAGE and transferred onto a PVDF membrane. After blocking with TBS buffer (20 mmol/L Tris–HCl, 150 mmol/L NaCl, pH 7.4) containing 5% nonfat milk, the membrane was incubated with monoclonal antibody followed by horseradish peroxidase-conjugated anti-mouse IgG, and then visualized using an ECL chemiluminescent detection kit (Amersham, Sweden).

### 3.6. Statistical Analysis

Values are expressed as means ± SD and analyzed using one-way ANOVA followed by LSD for comparisons of group means. All statistical analyses were performed using SPSS for Windows, version 10. Unless specified otherwise, a *P* < 0.05 is considered significant.
